# Genome-wide methylation profiling of the different stages of hepatitis B virus-related hepatocellular carcinoma development in plasma cell-free DNA reveals potential biomarkers for early detection and high-risk monitoring of hepatocellular carcinoma

**DOI:** 10.1186/1868-7083-6-30

**Published:** 2014-12-02

**Authors:** Yangxing Zhao, Feng Xue, Jinfeng Sun, Shicheng Guo, Hongyu Zhang, Bijun Qiu, Junfeng Geng, Jun Gu, Xiaoyu Zhou, Wei Wang, Zhenfeng Zhang, Ning Tang, Yinghua He, Jian Yu, Qiang Xia

**Affiliations:** State Key Laboratory of Oncogenes and Related Genes, Shanghai Cancer Institute, Renji Hospital, Shanghai Jiao Tong University School of Medicine, LN 2200/25,Xietu Road, Shanghai, 200032 China; Department of Liver Surgery, Ren Ji Hospital, School of Medicine, Shanghai Jiao Tong University, 160 Pujian Road, Shanghai, 200127 China; Zhongshan Hospital, Fudan University, 180 Fenglin Road, Shanghai, 200032 China; Ministry of Education Key Laboratory of Contemporary Anthropology School of Life Sciences, Fudan University, 220 Handan Road, Shanghai, 200433 China; Shanghai Cancer Institute,Renji Hospital, Shanghai Jiao Tong University School of Medicine, LN 2200/25,Xietu Road, Shanghai, 200032 China; Department of General Thoracic Surgery, Shanghai Chest Hospital, Shanghai Jiao Tong University, 241 West Huaihai Road, Shanghai, 200030 China; Key Laboratory of Contraceptive Drugs and Devices of NPFPC, Shanghai Institute of Planned Parenthood Research, 2140 Xietu Road, Shanghai, 200032 China

**Keywords:** Plasma, Cell-free DNA, HBV, HCC development, Genome-wide, DNA methylation

## Abstract

**Background:**

An important model of hepatocellular carcinoma (HCC) that has been described in southeast Asia includes the transition from chronic hepatitis B infection (CHB) to liver cirrhosis (LC) and, finally, to HCC. The genome-wide methylation profiling of plasma cell-free DNA (cfDNA) has not previously been used to assess HCC development. Using MethylCap-seq, we analyzed the genome-wide cfDNA methylation profiles by separately pooling healthy control (HC), CHB, LC and HCC samples and independently validating the library data for the tissue DNA and cfDNA by MSP, qMSP and Multiplex-BSP-seq.

**Results:**

The dynamic features of cfDNA methylation coincided with the natural course of HCC development. Data mining revealed the presence of 240, 272 and 286 differentially methylated genes (DMGs) corresponding to the early, middle and late stages of HCC progression, respectively. The validation of the DNA and cfDNA results in independent tissues identified three DMGs, including ZNF300, SLC22A20 and SHISA7, with the potential for distinguishing between CHB and LC as well as between LC and HCC. The area under the curve (AUC) ranged from 0.65 to 0.80, and the odds ratio (OR) values ranged from 5.18 to 14.2.

**Conclusions:**

Our data revealed highly dynamic cfDNA methylation profiles in support of HBV-related HCC development. We have identified a panel of DMGs that are predictive for the early, middle and late stages of HCC development, and these are potential markers for the early detection of HCC as well as the screening of high-risk populations.

**Electronic supplementary material:**

The online version of this article (doi:10.1186/1868-7083-6-30) contains supplementary material, which is available to authorized users.

## Background

Human hepatocellular carcinoma (HCC) is one of the most common primary liver malignancies; it is ranked fifth in incidence and third in mortality among common solid tumors worldwide [1, 2]. The highest incidences are observed in eastern Asia, where a model of multi-stage carcinogenesis that includes progression from chronic HBV infection (CHB) to HBV-related liver cirrhosis (LC) and, finally, to HBV-related HCC (HBV-HCC) has been suggested by both epidemiologic data and laboratory investigations [3, 4]. Three mechanisms are involved in HBV-related carcinogenesis, including the following: (1) the interruption of proper liver cell proliferation and viability from HBV proteins, especially HBx [5]; (2) changes in gene function and instability, which are induced by the insertion of viral DNA into the host genome; and (3) genomic and epigenomic injuries resulting from liver cell inflammation that are induced by immune T cell targeting the HBV virus [6].

Even in the early stages of chronic hepatitis B infection, DNA methylation (DNAm) appears to be altered, and some of these aberrant methylation patterns could overlap with those that are associated with HCC. Some studies have reported that the host cell modifies foreign HBV DNA by methylation and histone acetylation, hindering viral RNA replication and protein expression as defense mechanisms against viral invasion. However, the enhanced expression of the host DMNT also leads to the methylation of its own CpG island (CGI), particularly for those that are located in the promoter regions of tumor suppressor genes (TSG), such as p16INK4A, RASSF1A, GSTPI and MGMT as well as some miRNAs [7, 8]. Previous reports have demonstrated that HBV products, including the HBx protein, may manipulate DNMT1 to inactivate some TSGs, such as CDH1, by the dysregulating methylation or by aberrantly methylating miRNAs to influence downstream targets, such as DNMT3A, leading to further disturbances in methylation [9].

Previous reports have shown that DNA methylation is a multi-step event, which is similar to tumorigenesis [10]. Therefore, the establishment of DNA methylation profiles at the whole genome level, including those from healthy control (HC) livers and CHB, LC and HCC samples, will provide fundamental information about the common or unique DNA methylation patterns for different stages of HCC development, which may even facilitate the identification of the genes that are involved in this process.

To date, approximately five studies have reported the DNA methylation (DNAm) profiles of HCC tumor/adjacent tissues using Illumina arrays [11–15]. These studies have different sample sizes, technology and major etiologic causes (that is, HBV, HCV or alcohol). However, relatively little is currently known about DNA methylation alterations that occur at the early stages of HCC development, including those that are involved in the processes of CHB and LC. Additionally, genome-wide methylation profiling has not previously been reported for these two pathologic models. Moreover, the invasive procedure of tissue sampling limits the utility of this procedure for epidemiologic studies. In fact, CHB and LC samples are usually used as controls in genomic methylation analyses of HCC or in-gene locus-specific CGI studies [16].

However, the biggest challenge in studying DNA methylation profiling of distinct HCC stages is the impossibility of accessing the corresponding primary liver tissues. In contrast, blood sampling is superior to tissue sampling because it is minimally invasive, easy to obtain and can be consecutively applied as serial samples in clinical practice. Research shows that there is only a tiny level of cell-free DNA (cfDNA) in peripheral blood circulation in healthy individuals, which substantially increases during the development of HCC, when high levels of cfDNA are released into the bloodstream from apoptotic or necrotic HCC cells. It has been reported that increased levels of cfDNA are released into the blood in cases of hepatitis or cirrhosis compared with those resulting from HCC [17]. cfDNA is typically utilized for gene locus-specific DNA methylation analyses, whereas the genomic methylation profiling of cfDNA has seldom been reported. However, the accurate identification of primary tumor genomic methylation patterns in plasma cfDNA has recently been confirmed in a study on esophageal cancer [18].

**Figure 1:**
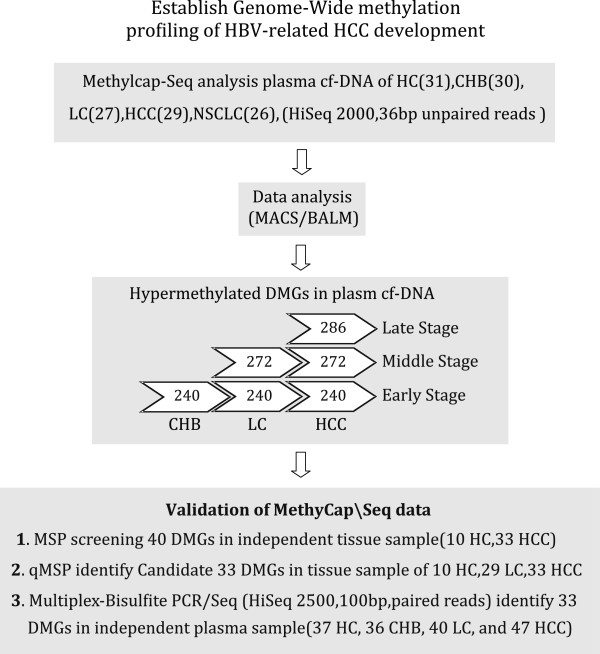
**Overview of the experimental strategy for evaluating differential DNA methylation in various stages of hepatocellular carcinoma development.** HC, healthy control; CHB, chronic hepatitis B virus (HBV)-related hepatitis; LC, HBV-related liver cirrhosis; HCC, HBV-related hepatocellular carcinoma (number in parentheses: participant).

In the present study, we conducted a genome-wide screening analysis to detect aberrant methylation events in plasma cfDNA using the MethylCap-seq method, and we observed some distinct DNAm alterations that were associated with the CHB, LC and HCC developmental stages (Figure 1). These findings enhance our understanding of the epigenetic disturbances in HCC plasma and provide novel tools for assessing high-risk populations for the presence of this disease.

## Results

### Genome-wide methylation profiling with plasma cell-free DNA from different stages of hepatitis B virus-related hepatocellular carcinoma development

**Figure 2:**
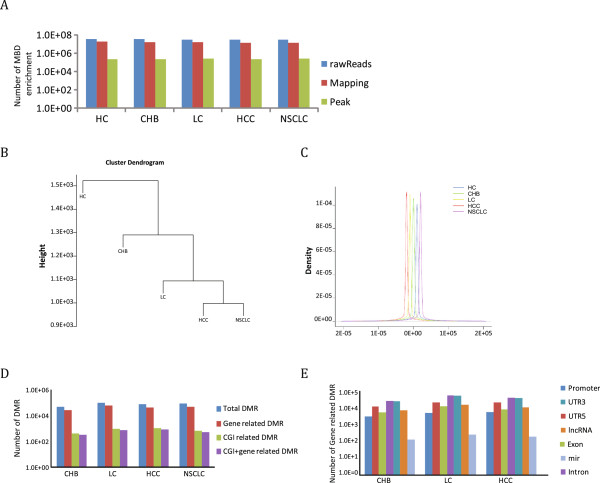
**Data mining and overall features of the MethylCap-seq libraries of plasma cfDNA during hepatocellular carcinoma (HCC) development. (A)** Analysis of the MethylCap-seq library generated 37,610,900; 37,072,952; 35,016,215 or 33,286,609 and 33,002,633 raw reads for health control (HC), chronic hepatitis B infection (CHB), liver cirrhosis (LC), or HCC and non-small cell lung cancer(NSCLC) (blue bars), respectively. Nearly half of the raw reads (43.3-50.7%) could be mapped to the hg19 reference genome (brown bar), producing 180,000-260,000 methylation peaks (green bar). **(B)** Cluster analysis based on genome-wide DNA methylation similarities. Hierarchical clustering was conducted to show the similarities in the DNAm among CHB, LC, HCC and NSCLC. The Euclidean distance was applied to measure the similarities in methylation alterations. Hypermethylated regions were defined as the regions in which methylated reads were over threefold higher than those observed in normal tissues through a 100-kb sliding window and 50-kb steps. **(C)** The density distribution of DNA methylation near the transcription start site (TSS). Alterations in DNAm were surveyed over a broad region of the gene (from 200 kb downstream to 200 kb upstream of the TSS). HCC had the highest levels of methylation in the TSS region, which was followed by LC, CHB and HC. As a control, NSCLC was plotted in the figure to show that HCC and NSCLC had the highest levels of methylation near the TSS. The original overlapping peaks are artificially separated to enable clear views of each peak. **(D)** Differential methylated regions (DMR) obtained by category, including the total DMR, gene-related DMR, CGI-related DMR and both CGI- and gene-related DMR. **(E)** General characterizations of the gene structures as determined by aberrantly methylated genomic loci in the different stages of HCC development (CHB, LC and HCC). The Y-axis depicts the hit numbers of the genes.

Data mining resulted in 37,610,900; 37,072,952; 35,016,215; 33,286,609 and 33,002,633 raw reads for the samples from the HC, CHB, LC, HCC, and NSCLC patients, respectively. When these were mapped to the hg19 genome reference, 19,083,142; 18,285,110; 16,389,041; 14,422,110 and 14,894,363 reads were obtained, respectively, with a mapping rate of approximately 50% (Figure 2A, Additional file 1: Table S4). Notably, very high background levels of methylation were observed for the HC plasma cfDNA.

### Association of plasma cell-free DNA methylation disturbances with the progressive stages of hepatocellular carcinoma development

We used hierarchical clustering to trace the progression of methylation disturbances during the carcinogenic process for the HC, CHB, LC and HCC samples. The analysis revealed the closeness of aberrant DNA methylation patterns among different groups of samples (Figure 2B), suggesting the possible sequential direction of the development of epigenetic alterations for the samples. Moreover, we estimated the relationships between the aberrant methylation patterns in the various stages of HCC development by the pairwise overlap of the peak bases from each stage, which revealed the similarities between HC and CHB as well as between LC and HCC (Additional file 2: Table S5). In addition, we investigated the DNA methylation densities surrounding the transcription start site (TSS), which is a crucial region that affects the gene expression of regulatory mechanisms, and we observed an accumulation of aberrant DNA methylation events as the disease stage progressed from HC to CHB to LC and, finally, to HCC (or NSCLC) (Figure 2C). These results suggest a stage-related stepwise process in which the gene methylation regulatory elements were sequentially affected during the carcinogenesis process. Collectively, the above observations indicate that the aberrant methylation of plasma cfDNA occurs in a dynamic, progressive and stage-responsive manner during HCC development. This creates the basis for our further analyses of HCC progression-related DNA methylation abnormalities.

### Identification of differentially methylated loci among the various stages of hepatocellular carcinoma development

To reliably obtain the DNA methylation peaks, we used two methods, MACS and BALM. The peaks that were generated by these two methods were generally consistent (90 to 97% coincidence). We performed our analysis using these peaks, which totaled 226,898; 257,344; 282,203; 242,987 and 290,096 for the HC, CHB, LC, HCC and NSCLC groups, respectively (Additional file 3: Table S6).

Because our long-term aim was to establish methylation markers that can be used for detecting and monitoring HCC development and progression, we were particularly interested in hypermethylated DNA alterations. To identify the stage-related DNA methylation disturbances, we investigated the alterations at each stage, including CHB, LC and HCC as well as NSCLC, which were compared to the normal background levels of the HC. The different methylation peaks that were subsequently generated are referred to as the differential methylated regions (DMRs). The most abundant DMRs were observed in the “total DMR” category, reflecting all of the hypermethylation peaks that were elevated compared with the HC (Figure 2D; Additional file 3: Table S6). In addition, the “gene-related DMR” and “CGI-associated DMR” categories could be isolated, and the category that included both the CGI and gene-related DMRs became the focus of our further analyses (Figure 2D, Additional file 4: Table S7).

The analysis of aberrantly methylated DMRs at different loci in association with the HCC developmental process also revealed that widespread genomic structural elements were affected, which included a variety of structural elements as defined by the UCSC Genome Bioinformatics (UCSC) (Figure 2E and Additional file 5: Table S8).

### Early-, middle- and late-stage differentially methylated regions and their related differentially methylated genes

**Figure 3:**
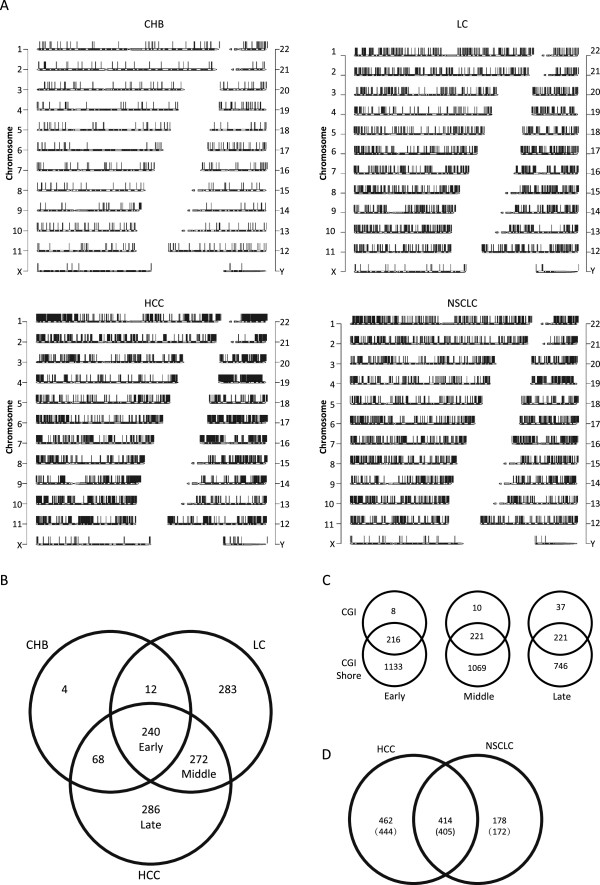
**Definition and selection of stage-specific differentially methylated region (DMR) during hepatocellular carcinoma (HCC) development. (A)** Chromosomal views of the genome-wide methylation statuses of chronic hepatitis B infection(CHB), liver cirrhosis (LC) and hepatocellular carcinoma (HCC) and non-small cell lung cancer(NSCLC). Black bars represent hypermethylation relative to HC. DNA hypermethylation occurs as early as the CHB stage and becomes more extensive as HCC development progresses. NSCLC, an alternative non-liver malignancy included in this study to test effects of different organs on cfDNAm. **(B)** Venn diagram of the hyper-differentially methylated genes (DMGs) that occurred in the CHB, LC and HCC stages. The diagram was used to further analyze the relationships among these three groups. **(C)** Gene mappings based on DMG information. To expand upon the identification of genes that are affected during the early, middle and late stages in the DMRs, genes that were located proximal to and involved in the hypermethylation events, such as “CGI only”, “both CGI and shore” or “shore only”, were examined. Here, “shore” is defined as a region that flanks traditional CGIs (up to 2 kb in distance). **(D)** Venn diagram depicting the comparison of the hyper-DMGs that occurred in HCC versus NSCLC. In total, 405 hyper-DMGs are shared by HCC and NSCLC, indicating the general homogeneities of the different malignancies, such as HCC and NSCLC in this case.

To further elucidate the cfDNA methylation alterations that occur during the CHB, LC and HCC stages, we compared the DMRs that were associated with each stage on a chromosomal distribution basis against those from the HC (Figure 3A). The analysis showed that CHB is characterized by sparse and mild hypermethylation alterations, suggesting that certain HBV-induced epigenetic disturbances begin as early as this stage. However, these hypermethylation alterations obviously increased during the transition from CHB to LC, indicating the presence of more extensive epigenetic disturbances. The HCC stage was characterized by substantial hypermethylation, and a similar degree of epigenetic change was also observed for NSCLC, suggesting that such alternations are commonly associated with various malignancies in the late stages of carcinogenesis.

Provided that the introduction of DMRs was a continuous, dynamic process during the different stages of HCC development, we observed the following three categories of DMR involvement: 1) those that were common to CHB, LC and HCC, which may play important roles in the process of HCC development from CHB to HCC; because they occurred as early as the CHB stage, we referred to them as early DMRs; 2) those that were common to both LC and HCC, which may be essential for the transition from LC to HCC; we called these middle DMRs; and 3) those that were unique to HCC, which are most likely indispensible for maintaining the malignant status; we called these late DMRs. To identify the exact genes that are affected by the aforementioned early-, middle- and late-stage DMRs (the differentially methylated genes (DMGs)), we used “CGI + gene” involvement as the selection criterion and generated 240 early-, 272 middle- and 286 late-stage DMGs (Figure 3B, Additional file 6: Table S9, Additional file 7: Table S10 and Additional file 8: Table S11).

Recent studies have reported that tissue and cancer-specific DMRs occur more frequently within CGI shores, which are regions of relatively low CpG densities that flank traditional CGIs (up to 2 kb in distance) than within CGIs themselves, indicating the importance of considering CGI shores in DNA methylome analyses [19–21]. Therefore, we generated 225 early-, 232 middle- and 259 late-stage DMGs according to CGI involvement (Additional file 9: Table S12, Additional file 10: Table S13 and Additional file 11: Table S14), while a significantly expanded panel of 1,349 early-; 1,290 middle- and 967 late-stage DMGs were generated when CGI-shore involvement was considered (Figure 3C, Additional file 12: Table S15, Additional file 13: Table S16 and Additional file 14: Table S17). This represents an obvious trend where CGI-associated DMGs gradually increase along with HCC progression, while CGI-shore-associated DMGs decrease, possibly suggesting that the epigenetic effects that occur during the late stages of HCC development are associated with CGI alone rather than CGI-shores.

### Differential methylation profiling between hepatocellular carcinoma and non-small cell lung cancer

To locate plasma DNA methylation loci that are specific to HCC and that may serve as potential diagnostic markers, we compared the DMGs between HCC and NSCLC and found that 414 were shared by both malignancies, while 462 were specific to HCC and 178 were specific to NSCLC (Figure 3D and Additional file 15: Table S18, Additional file 16: Table S19 and Additional file 17: Table S20). These results suggest that although carcinogenesis and tumor development may originate from different tissues, they share common DNA methylation alterations, as well as possess unique disturbances. Therefore, these differences can be used to determine the different tissue origins of carcinomas.

### Functional involvement of plasma cell-free DNA differentially methylated genes associated with hepatocellular carcinoma developmental stages

We performed gene ontology and pathway analyses of the early-, middle- and late-stage HCC DMGs using the DAVID method. All of these DMGs were selected for analysis because their promoter regions were aberrantly methylated.

Initially, we analyzed 191 early-stage DMGs. According to the results, 36 GO terms were enriched in association with hypermethylation using this set of DMGs. The top ten GO terms included the bioprocesses of “cell adhesion” (four terms), “cyclase activity” (two terms), “cytoskeleton organization” (one term), “synaptic transmission” (one term), “cAMP biosynthetic process” (one term) and “lyase activity” (one term). Our pathway analysis revealed the presence of three methylation-enriched processes, including “neuroactive ligand-receptor interaction”, “androgen and estrogen metabolism” and “steroid hormone biosynthesis” (Tables 1 and 2). Furthermore, we characterized the GO terms for the middle-stage DMGs and found that the 209 DMGs were associated with 71 GO terms. The top ten included “cell adhesion’ (two terms), ‘nervous system’ (three terms), ‘collagen catabolism’ (one term), ‘hormone levels’ (two terms), ‘cell-cell signaling’ (one term) and ‘regulation of secretion’ (one term). We found four pathways that correlated with these DMGs, which included the ‘calcium signaling pathway’, ‘pathway in cancer’, ‘basal cell carcinoma’ and ‘neuroactive ligand-receptor interaction’ (Tables 1 and 2). Ultimately, we found that a total of 231 late-stage DMGs, which were representative of the advanced malignant process of hepatic neoplasia, resulted in 93 GO terms. The top ten included ‘cell adhesion’ (four terms), ‘regionalization’ (one term), ‘developmental process’ (two term), ‘regulation of transcription’ (two terms) and ‘regulation of RNA metabolism’ (one term). In this late stage, the two most enriched KEGG pathways were ‘neuroactive ligand-receptor interaction’ and ‘pathway in cancer’ (Tables 1 and 2).

**Table 1:** **GO function analysis of the DMGs in plasma cf-DNA in different stage of HCC development**

Stage	Term	Count	%	PValue	Genes
**Early**	GO:0007156 ~ homophilic cell adhesion	15	7.85	6.63E-11	PCDHGA1,PCDHGA2,PCDHA3,PCDHB5,PCDHA4,PCDHGA3,PCDHGA5,PCDHB12,PCDHB4,PCDHGB2,PCDHGA4,PCDHGB1,PCDH17,PCDHA5,PCDHA2
	GO:0016337 ~ cell-cell adhesion	16	8.38	1.53E-07	PCDH17,PCDHA2,PCDHA3,PCDHA4,PCDHA5,PCDHB5,ICAM4,PCDHB4,PCDHB12,PCDHGA5,PCDHGA4,PCDHGA3,PCDHGA2,PCDHGA1,PCDHGB1,PCDHGB2
	GO:0007155 ~ cell adhesion	19	9.95	2.94E-04	PCDH17,PCDHA2,PCDHA3,PCDHA4,BCAR1,PCDHA5,PCDHB5,ICAM4,PCDHB4,PCDHB12,PCDHGA4,PCDHGA3,PCDHGA2,PCDHGA1,PCDHGB1,LOXL2,PCDHGB2,TNXB
	GO:0022610 ~ biological adhesion	19	9.95	2.99E-04	PCDH17,PCDHA2,PCDHA3,PCDHA4,BCAR1,PCDHA5,PCDHB5,ICAM4,PCDHB4,PCDHB12,PCDHGA5,PCDHGA4,PCDHGA3,PCDHGA2,PCDHGA1,PCDHGB1,LOXL2,PCDHGB2,TNXB
	GO:0007010 ~ cytoskeleton organization	14	7.33	5.53E-04	MACF1,CDC42BPG,PYY,BCAR1,TCHH,ACTA1,STMN1,GRID2IP,FSCN2,PRKCZ,CYTH2,NEFH,TNXB,SYNM
	GO:0045761 ~ regulation of adenylate cyclase activity	6	3.14	3.08E-03	HTR1A,HTR7,GNAZ,GIPR,GABBR1,ADRB1
	GO:0031279 ~ regulation of cyclase activity	6	3.14	3.52E-03	HTR1A,HTR7,GNAZ,GIPR,GABBR1,ADRB1
	GO:0007268 ~ synaptic transmission	10	5.24	3.65E-03	HTR7,PCDHB5,PCDHB4,DLGAP2,KCNC4,GRIK4,LIN7B,MTNR1B,SLC1A6,KCNN3
	GO:0030817 ~ regulation of cAMP biosynthetic process	6	3.14	3.84E-03	HTR1A,HTR7,GNAZ,GIPR,GABBR1,ADRB1
	GO:0051339 ~ regulation of lyase activity	6	3.14	3.84E-03	HTR1A,HTR7,GNAZ,GIPR,GABBR1,ADRB1
**Middle**	GO:0007156 ~ homophilic cell adhesion	7	3.35	2.95E-03	PCDH17,DCHS1,PKD1,PCDHGA3,PCDHGA2,PCDHGA1,PCDHGB1
	GO:0019226 ~ transmission of nerve impulse	10	4.78	1.37E-02	KCNMB3,NPBWR1,OXT,VIPR1,CHAT,SPTBN4,EGR3,SLC12A5,SLC17A7
	GO:0030574 ~ collagen catabolic process	3	1.44	1.94E-02	MMP11,MMP9,PRTN3
	GO:0007628 ~ adult walking behavior	3	1.44	2.32E-02	TRH,SPTBN4,CHAT
	GO:0010817 ~ regulation of hormone levels	6	2.87	2.39E-02	SHH,RBP1,DUOX1,FOXE1,SMPD3,ECE2
	GO:0030900 ~ forebrain development	6	2.87	2.45E-02	ID4,LHX6,SHH,ZIC5,AVPR1A,FOXG1
	GO:0042445 ~ hormone metabolic process	5	2.39	2.77E-02	SHH,RBP1,DUOX1,FOXE1,ECE2
	GO:0007267 ~ cell-cell signaling	13	6.22	2.91E-02	Sep5,SLC17A7,SHH,TRH,EGR3,SMPD3,ECE2,SLC12A5,OXT,NPBWR1,VIPR1,TNFSF9,CHAT
	GO:0016337 ~ cell-cell adhesion	8	3.83	3.01E-02	PCDHGA1,SCARF2,PKD1,PCDHGA3,DCHS1,PCDH17,PCDHGA2,PCDHGB1
	GO:0051047 ~ positive regulation of secretion	5	2.39	3.03E-02	Sep5,TRH,OXT,SCAMP5,AVPR1A
**Late**	GO:0007156 ~ homophilic cell adhesion	34	14.72	1.99E-33	PCDHGA5,PCDHGA12,PCDHA9,PCDHA10,PCDHA13,PCDHAC1,RET,PCDHGA6,PCDHGB7,PCDHGB6,PCDHA7,PCDHGB5,PCDHA5,PCDHGA1,PCDHGB2,PCDHA1,PCDHA4,PCDHB11,PCDH7,PCDHGA3,PCDHGA8,PCDHGA4,PCDHGA2,PCDHGB4,PCDHGA9,PCDHB16,PCDHGA10,PCDHA3,PCDHGA11,PCDHA11,PCDHGA7,PCDHGB1,PCDHA2,PCDHGB3,PCDHA6,PCDHA8,PCDH8,PCDHA12
	GO:0016337 ~ cell-cell adhesion	37	16.02	9.80E-26	PCDHGA5,PCDHGA12,SCARF2,PCDHA9,PCDHA10,PCDHA13,PCDHAC1,RET,PCDHGA6,PCDHGB7,PCDHGB6,PCDHA7,PCDHGB5,PCDHA5,PCDHGA1,PCDHGB2,PCDHA1,PCDHA4,PCDHB11,PCDH7,PCDHGA3,PCDHGA8,PCDHGA4,PCDHGA2,PCDHGA9,PCDHGB4,PCDHB16,PCDHGA10,PCDHA3,PCDHGA11,PCDHA11,PCDHGA7,PCDHGB1,PCDHA2,PCDHGB3,CALCA,PCDHA6,PCDHA8,PCDH8,CLDN11,PCDHA12
	GO:0007155 ~ cell adhesion	46	19.91	2.45E-19	PCDHGA5,ITGBL1,PCDHGA12,SCARF2,PCDHA9,PCDHA10,PCDHA13,PCDHAC1,RET,PCDHGA6,PCDHGB7,PCDHGB6,COL5A3,PCDHA7,PCDHGB5,PCDHA5,PCDHGA1,PCDHA1,PCDHA4,PCDHGB2,NLGN4X,PCDHB11,PCDH7,PCDHGA3,PODXL2,PCDHGA8,MAGI1,PCDHGA4,PCDHGA2,PCDHGA9,PCDHGB4,COL16A1,PCDHB16,ACHE,PCDHGA10,PCDHA3,GP5,PCDHGA11,PCDHA11,PCDHGA7,PCDHGB1,PCDHA2,PCDHGB3,COL18A1,CALCA,PCDHA6,PCDHA8,PCDH8,CLDN11,PCDHA12
	GO:0022610 ~ biological adhesion	46	19.91	2.59E-19	PCDHGA5,ITGBL1,PCDHGA12,SCARF2,PCDHA9,PCDHA10,PCDHA13,PCDHAC1,RET,PCDHGA6,PCDHGB7,PCDHGB6,COL5A3,PCDHA7,PCDHGB5,PCDHA5,PCDHGA1,PCDHA1,PCDHA4,PCDHGB2,NLGN4X,PCDHB11,PCDH7,PCDHGA3,PODXL2,PCDHGA8,MAGI1,PCDHGA4,PCDHGA2,PCDHGA9,PCDHGB4,COL16A1,PCDHB16,ACHE,PCDHGA10,PCDHA3,GP5,PCDHGA11,PCDHA11,PCDHGA7,PCDHGB1,PCDHA2,PCDHGB3,COL18A1,CALCA,PCDHA6,PCDHA8,PCDH8,CLDN11,PCDHA12
	GO:0003002 ~ regionalization	13	5.63	1.28E-05	PAX1,LHX1,TCF15,GATA4,TBX20,HOXC4,CYP26B1,TBX3,GBX2,PCDH8,MYF6,CYP26C1,HOXD4
	GO:0007389 ~ pattern specification process	15	6.49	1.34E-05	PAX1,LHX1,TCF15,ZIC1,GATA4,TBX20,BCOR,HOXC4,CYP26B1,TBX3,GBX2,PCDH8,MYF6,CYP26C1,HOXD4
	GO:0009952 ~ anterior/posterior pattern formation	11	4.76	1.75E-05	PAX1,HOXC4,LHX1,GBX2,TBX3,PCDH8,TCF15,MYF6,CYP26C1,HOXD4,GATA4
	GO:0006355 ~ regulation of transcription, DNA-dependent	44	19.05	5.34E-05	PATZ1,PRDM16,PAX1,LHX1,SIM1,IRX2,ZNF148,TBX2,PHOX2B,FOXP4,TWIST2,CDKN2A,EVX2,ZNF808,LHX9,TLX1,FOXO3,FOXO3B,ELAVL2,VSX1,TBX3,GBX2,SPI1,BMP2,ISL2,IRX5,HOXD4,MEIS1,EBF3,TCF15,ZNF619,DMRT1,KCNH6,SOX1,TBX20,SOX7,ZNF560,GATA4,L3MBTL4,HOXC4,BCOR,RFX1,MYF6,TBX4,NPAS4
	GO:0051252 ~ regulation of RNA metabolic process	44	19.05	9.00E-05	PATZ1,PRDM16,PAX1,LHX1,SIM1,IRX2,ZNF148,TBX2,PHOX2B,FOXP4,TWIST2,CDKN2A,EVX2,ZNF808,LHX9,TLX1,FOXO3,FOXO3B,ELAVL2,VSX1,TBX3,GBX2,SPI1,BMP2,ISL2,IRX5,HOXD4,MEIS1,EBF3,TCF15,ZNF619,DMRT1,KCNH6,SOX1,TBX20,SOX7,ZNF560,GATA4,L3MBTL4,HOXC4,BCOR,RFX1,MYF6,TBX4,NPAS4
	GO:0045449 ~ regulation of transcription	55	23.81	3.16E-04	PRDM16,PAX1,SIM1,IRX2,TBX2,PHOX2B,FOXP4,ZNF808,TBX3,GBX2,SPI1,HOXD4,MEIS1,APBB2,PPP1R13L,TCF15,ARID3C,NRK,SOX1,TBX20,SOX7,CALCA,HIVEP3,TBX4,PATZ1,LHX1,ZNF148,ZNF703,TWIST2,CDKN2A,EVX2,FOXO3,FOXO3B,TLX1,LHX9,ELAVL2,VSX1,BMP2,ISL2,IRX5,EBF3,FERD3L,ZNF619,DMRT1,KCNH6,SCRT2,GATA4,ZNF560,L3MBTL4,BCOR,HOXC4,AMH,RFX1,INSM2,MYF6,NPAS4

**Table 2:** **KEGG pathway analysis of the DMGs in plasma cf-DNA in different stage of HCC developmen**

Stage	Term	Count	%	PValue	Genes
**Early**	hsa04080:Neuroactive ligand-receptor interaction	9	4.71	1.35E-03	HTR1A,S1PR5,HTR7,GIPR,GRIK4,P2RY6,MTNR1B,GABBR1,ADRB1
	hsa00150:Androgen and estrogen metabolism	3	1.57	4.02E-02	LCMT2,SRD5A3,SULT2B1
	hsa00140:Steroid hormone biosynthesis	3	1.57	5.96E-02	CYP1B1,SRD5A3,SULT2B1
**Middle**	hsa04020:Calcium signaling pathway	6	2.87	2.61E-02	TNNC2,AVPR1A,ITPR3,PDE1C,BST1,ADRB3
	hsa05200:Pathways in cancer	7	3.35	9.31E-02	RARA,SHH,GSTP1,PAX8,WNT10B,MMP9,APC2
	hsa05217:Basal cell carcinoma	3	1.44	9.79E-02	SHH,WNT10B,APC2
	hsa04080:Neuroactive ligand-receptor interaction	6	2.87	9.82E-02	GPR35,AVPR1A,NPBWR1,VIPR1,ADRB3,S1PR5
**Late**	hsa04080:Neuroactive ligand-receptor interaction	9	3.9	1.15E-03	HTR2C,GABRG3,MLNR,GPR83,ADRA1A,S1PR4,GALR2,ADORA3,ADRB3
	hsa05200:Pathways in cancer	8	3.46	1.88E-02	FGF8,CDKN2A,WNT10A,RET,SPI1,BMP2,MMP9,BCR

### Methylation-specific PCR (MSP) screening, qMSP validation and gene expression of the library revealed differentially methylated genes in independent tissue DNA

**Figure 4:**
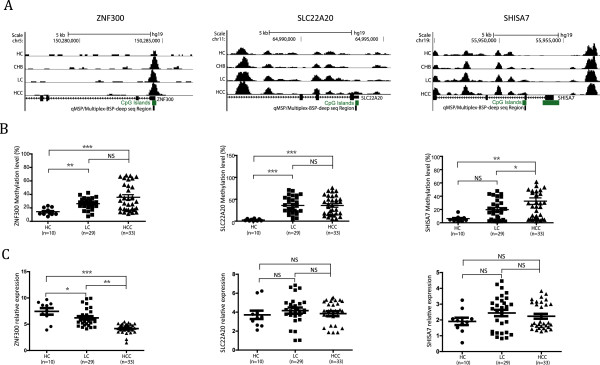
**Quantitative methylation-specific PCR (qMSP) and gene expression test of MethylCap-seq library data using tissue DNA and RNA templates.** The screening analysis generated a set of 33 genes that are informative of hepatocellular carcinoma (HCC). This set of genes was further analyzed by qMSP and gene expression using real-time PCR in DNA and RNA samples from 10 health control (HC), 29 liver cirrhosis (LC) and 30 HCC tissues. Three representative gene results are shown here. **A)** The UCSC scheme of the gene locus and examined promoter region are shown as well as the methylation information in HC, chronic hepatitis B infection (CHB), LC and HCC groups. **B)** Quantification of the methylation status in HC, LC and HCC. **C)** Relative gene expression assay by RT-PCR in HC, LC and HCC. For statistical significance, *P <0.05; **P <0.01; ***P <0.001; NS, not significant.

Two techniques were used to assess the tissue DNA for validating the accuracy of the plasma DNAm profile library. First, 40 DMR targets that possessed the top *P* values were screened by MSP in 10 normal liver and 33 HCC (Additional file 18: Figure S2) tissue DNA samples, generating 33 targets that were informative of HCC. Subsequently, the informative targets were investigated by qMSP in the same tissues sets that had supplemented with 29 LC tissues. As expected, a substantial portion of the targets exhibited hypermethylation in the LC, HCC or both the LC and HCC. The typical results in selected genes are shown in Figures 4A and B, with the UCSC scheme of the gene information and their quantitative methylation statues in HC, LC and HCC. These results suggest that the altered methylation patterns that were observed in the plasma are consistent with the respective liver tissues.

In addition, the expression levels of selected genes, ZNF300, SLC22A20 and SHISA7, were examined using real-time reverse transcription (RT-PCR) in the liver tissue of HC, LC and HCC. There appeared a coincidence that hypermethylated ZNF300 showed downregulation with progression of HC to LC and then to HCC (*P* <0.05). However, SLC22A20 and SHISA7 did not have hypermethylation coupled with gene silence pattern (Figure 4C).

### Multiplex-BSP-seq validation of differentially methylated genes in independent plasma cell-free DNA

**Figure 5:**
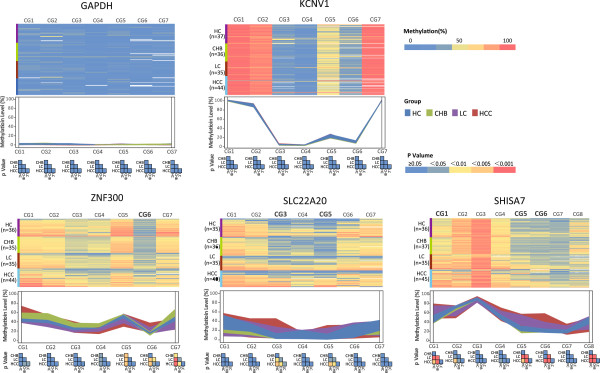
**Representative results of the quantitations of the methylation levels by Multiplex-BSP-seq for health control (HC), chronic hepatitis B infection(CHB), liver cirrhosis (LC) and hepatocellular carcinoma (HCC) plasma DNA samples.** For each gene, the heat map of the methylation patterns for each CpG is shown. The methylation level (%) measured at each individual CpG site is expressed by the percentage of methylated CpG versus unmethylated CpG sites. HC, CHB, LC and HCC are represented by colored areas of blue, green, violet and red, respectively. The colored area is defined by 25%/75% quantiles. The comparison of the CpG methylation levels for each stage is colored blue to red, in small squares, to indicate the different *P* values. Representative heat map and methylation plot analysis of 5 target genes; consistently low levels of methylation of all CpGs were observed in GAPDH and steady high levels of CpG(CG1,2 and 7) methylation in KCNV1 are independent of the HCC developmental stage, while the methylation statuses of the other 3 genes (ZNF300, SLC22A20 and SHISA7) varied according to the developmental stage.

**Figure 6:**
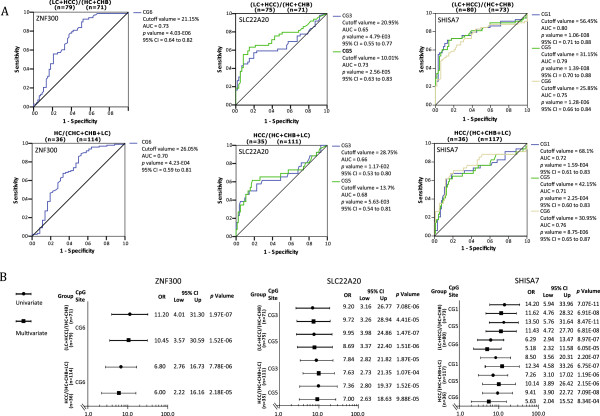
**Receiver operating characteristics (ROC) and multiple univariate logistic regression analyses for using CpGs to distinguish between hepatocellular carcinoma (HCC) developmental stages are shown according to the CpG position and disease stage (HC + CHB versus LC + HCC or HC + CHB + LC versus HCC). (A)** Receiver operating characteristic (ROC) curves for ZNF300, SLC22A20 and SHISA7. Complete DNA methylation data from all four stages of HCC development were used to construct the ROC curves. The ROC curves plot the sensitivity versus 100-specificity. Upper panel: a lower cut-off value was used to distinguish between (LC + HCC)/ (HC + CHB).Lower panel: a higher cut-off value was used to distinguish between HCC/(HC + CHB + LC). **(B)** A multiple univariate logistic regression analysis was performed using the CpG methylation patterns to evaluate the association between gene methylation and the stage of HCC development. Relationship between the CpG methylation (odds ratios) and the developmental stage. To separate (LC + HCC)/ (HC + CHB) and HCC/(HC + CHB + LC), both univariate (which considers the methylation levels) and multivariate (which also considers the age and gender) logistic regressions were performed using CpG methylation data for ZNF300, SLC22A20 and SHISA7.

To further confirm the cfDNA methylation levels, we used Multiplex-BSP-seq on the targets that were selected by qMSP in the independent plasma cfDNA samples (Additional file 19: Table S2). Of the 33 target genes that were studied, 22 were successfully amplified and deep-sequenced. The representative results are shown in Figure 5. For each gene, the methylation levels of seven to ten individual CpG sites were investigated. GAPDH and KCNV1 exhibited relatively fixed methylation patterns for the seven CpGs that were inspected, regardless of the HCC developmental stage. Therefore, both can serve as quality control measurements. In this study, the methylation levels in some of the CpGs of 3 particular genes were closely related to the developmental stage of HCC; therefore, they were predictive of the disease stage. These genes and their informative CpG sites included ZNF300 at CpG6, SLC22A20 at CpG3 and 5 and SHISA7 at CpG1, 5 and 6 (see Figure 6A, B), which could distinguish between HC + CHB and LC + HCC with a lower cut-off value, while distinguishing between HC + CHB + LC and HCC with a higher cut-off value of CpG methylation (Figure 6A). When these CpG methylation measurements were subjected to multiple univariate logistic regression analyses, they showed strong or very strong associations with stage discrimination (OR: 5 to approximately 14) in distinguishing between HC + CHB and LC + HCC or between HC + CHB + LC and HCC (Figure 6B). Taken collectively, Multiplex-BSP-seq using an independent set of plasma samples validated certain CpG methylation changes in a subset of genes that were revealed by MethylCap-seq during HCC development. This also suggests the important role of aberrant methylation in these CpG sites during HCC carcinogenesis.

## Discussion

In the present study, methylCap-seq was applied to establish genome-wide methylation profiles in which a cross-sectional group of HC, CHB, LC and HCC patients was recruited for a process consequence model study. In addition, NSCLC was included as a non-liver malignancy group. Plasma cfDNA was collected from these individuals for the analyses. Because of the minute quantities of cfDNA that were obtained, MethylCap-seq analyses could not be performed individually; instead, the cfDNA was pooled. In fact, sample pooling has been used in a number of -omic studies because it is cost-efficient and suitable for scarce sample models [22, 23].

In this experiment, we initially explored the features of genome-wide DNA methylation profiling by cluster analyses and found similarities between the patterns of aberrant DNA methylation for the different HCC developmental stages, which resembled the natural course of this disease. Hence, our study model can be used to assess the patterns of DNA methylation alterations in cfDNA that underlie HCC development. We further compared our plasma data, which indicated significant levels of hypermethylation in HCC relative to HC, with data that on tissue DNA. Shen *et al.* [15] analyzed HCC tumor and adjacent non-tumor tissues from 62 Taiwanese HCC cases using Illumina methylation arrays and found that 684 CpG sites in 550 genes were significantly hypermethylated in the tumor tissues. In fact, 343 of the genes from that study were detected in our plasma HCC analysis (Additional file 20: Table S21) in spite of the different analysis methods and different samples used in both investigations. Therefore, these identical genes may serve as good resources for selecting plasma markers for HCC evaluations because they exhibit hypermethylation not only in the tissues but also in the cfDNA; therefore, the cfDNA methylation alterations parallel those of hepatic cells.

According to the accepted hypothesis, aberrant DNA methylation in association with carcinoma is characterized by genome-wide hypomethylation and clustered hypermethylation [24, 25]. Therefore, we analyzed the relationship between HCC stage-related DMRs and gene promoters and observed that the DMRs were more abundant in the promoters during the advanced stages of HCC development than in the early stages, and the highest concentrations were present in the promoter regions for HCC and NSCLC. In terms of HCC development progression, we found 240 early-, 272 middle- and 286 late-stage DMGs. These results indicate that HBV invasion led to hypermethylation in the genomic DNA of the hepatic cells, and some of these alterations could be maintained as late as the HCC stage, which is presumably because they are the important factors for HBV-related HCC development. In addition, these results suggest that the alterations gradually accumulated, rather than appearing all at once, during the HBV-related HCC progression. It is possible that the late-stage DMGs are the driving factors underlying, or are merely consequences of, the hepatic cell malignant transformations that occur during HCC development.

Knowledge of the functional involvement of aberrantly methylated genes is important to understanding the mechanisms that occur during HCC development. We performed GO and pathway analyses on DMGs from the early, middle and late stages of HCC development. The GO analysis of the early-stage DMGs revealed the prominence of the ‘cell-adhesion’ and ‘neuroactive ligand-receptor interaction’ pathways. In a previous study, the regulatory profile of the HBx gene was investigated, and six pathways were deregulated, including ‘neuroactive ligand-receptor interaction’ and ‘cell adhesion molecules’ [26]. Another study investigated hypermethylated genes in single hepatocytes from HBV-related HCC, and the top KEGG pathway was identified as the ‘neuroactive ligand-receptor interaction’ [27]. All of these results suggest the extraordinary importance of the ‘neuroactive ligand-receptor’ pathway in HCC carcinogenesis. With respect to the two sex hormone metabolism-related pathways, ‘steroid hormone biosynthesis’ is a direct target of HBx [26]. Moreover, HCC predominantly occurs in males [28], and these results suggest that the gender involvement may be mediated by epigenetic disturbances in the steroid hormone metabolism and that these alterations may occur in the early stages of HBV-HCC progression. The appearance of the GO terms for ‘cell adhesion’ and ‘neuroactive ligand-receptor interaction’ in the middle-stage and early-stage DMGs suggest that they play indispensable roles during the early and middle stages of HCC development. In addition to these two inherited items, some other GO terms, including ‘nerve system’, ‘collagen and hormone’ and ‘cell signaling,’ are possibly responsible for the extensive pathogenesis in liver cirrhosis. The ‘calcium signaling pathway’ in this stage was also identified in other similar studies [26, 27]. The analysis of late-stage DMGs revealed the prominence of ‘cell adhesion,’ which was the top GO term, but other categories, such as the ‘developmental process’, ‘regulation of transcription’ and ‘regulation of RNA metabolism,’ may also be tightly associated with the malignant growth potential of HCC. Notably, the ‘neuroactive ligand-receptor interaction’ pathway was identified throughout the developmental stages, emphasizing its persistent role in HBV-related carcinogenic processes. We further isolated the plasma DMGs that occurred in the early, middle and late stages of HBV-related HCC development, which may be involved in particular GO functions and pathways related to HCC.

The DNA methylation patterns that we observed may play important roles in HCC development and facilitate early tumor diagnosis, disease progression monitoring and the identification of high-risk individuals. To further understand these findings, we used a combination of Multiplex-BSP-seq, Illumina index adaptor techniques and deep sequencing to validate 33 DMG targets in approximately 160 cfDNA samples from four stages of HCC development. This resulted in the identification of three relevant genes (SHISA7, ZNF300 and SLC22A20) in association with ten CpG methylation statuses that were informative of HBV-related HCC development. Similar to the methylation patterns observed in the MethylCap-seq analysis, Multiplex-BSP-seq also revealed higher similarities between HC and CHB as well as between LC and HCC, suggesting close relationships between their pathogenic processes in terms of epigenetic involvement. Epigenetic changes occurred gradually from HC to CHB and more substantially from CHB to LC. The accumulation of alterations slows in the transition to the final malignant state of HCC, and the entire process is not likely to occur at a steady rate as indicated by this study.

Epigenetics plays a role through regulating the corresponding gene expression. In our present study, the increasing hypermethylaton of gene ZNF300, during HCC development, is coupled with decreased gene expression. However, the remaining two genes, SHISA7 and SLC22A20, failed to have this correlation. These results might suggest that only a subset of hypermethylated genes might play a role through the methylation-gene silence pathway, while most of the remaining hypermethylated genes influence tumor development by alternative mechanisms.

The ROC analysis of these ten CpGs revealed that LC and HCC could be distinguished from HC and CHB when a lower methylation cut-off value was used, while HCC could be effectively discriminated from HC, CHB and LC when a higher methylation cut-off value was adopted. Univariate and multivariate logistic regression analyses indicated the strong association of these CpG methylation sites with LC and HCC, suggesting that these aberrant DNA methylations may represent risk factors for HCC development. Other researchers have reported that the DNA methylation patterns of single CpG bases may be useful indicators of carcinoma risk and development [29].

With respect to the three aforementioned genes, SLC22A20, which is located on chromosome 11q13.1, belongs to a family of transmembrane proteins that function as organic anion transporters by accepting a variety of chemically unrelated endogenous and exogenous anions, including many popular drugs [30, 31]. To date, there no previous reports have described the association of this gene with malignant tumors. We speculate that the aberrant methylation of this gene leads to the dysregulation of its expression, which contributes to HCC development from the accumulation or depletion of anions or pH alterations in the microenvironments of the hepatic cells. ZNF300, which is located on chromosome 5q33.1, possesses an amino-terminal KRAB domain and 12 carboxyl-terminal C2H2 zinc finger motifs. ZNF300 is localized to the nucleus, and the KRAB domain of this protein has transcription repressor activities. These results suggest that ZNF300 is a ubiquitous transcription repressor in the nucleus [32]. Additionally, it has been reported to promote tumor development by modulating the NF-KB pathway, in which it plays an important role during tumor development in inflamed tissues [33]. The prevalence and molecular mechanisms underlying inflammation have been extensively studied because inflammation is a risk factor for a subset of tumors. Furthermore, the invasion of hepatic cells by HBV results in inflammation, which accompanies LC and may later progress to HCC, and the involvement of ZNF300 in this process requires further study. SHISA7, which is located on chromosome 19q13.42, belongs to the SHISA family, but its exact function is unknown. The high overlap of these three genes in LC and HCC in terms of their methylation levels suggests that they have important roles in the progression from LC to HCC, defining them as pro-oncogenes or HCC-driven genes; however, more investigations are needed to formulate definitive conclusions.

## Conclusions

In summary, our present study on serum cfDNA methylation provides the following hypothesis, which could be validated: chronic HBV infection of hepatic cells triggers a series of aberrant DNA methylations that accompany the entire process of HBV-related HCC carcinogenesis, including the gradual accumulation of abnormal DNA hypermethylation. The affected gene targets have the potential to be both diagnostic biomarkers and therapeutic targets for HCC treatment.

## Methods

### Patient samples

With informed consent and the approval of the Medical Institutional Review Board of Renji Hospital and Shanghai Chest Hospital, Shanghai Jiao Tong University, patient specimens were collected between 2010 and 2012. Three separate sets of patient samples were obtained to establish the genome-wide methylation profiles of the plasma cfDNA by MethylCap-seq, screen and identify candidate differential methylation regions (DMRs) in tissue DNA by MSP/qMSP and validate candidate DMRs in plasma cfDNA by Multiplex-BSP-seq. The first set of samples included 31 HC, 30 CHB, 27 LC and 29 HCC cases in addition to 26 non-small cell lung cancer (NSCLC) samples, which were included as a control, representing a malignancy from a separate organ. The second set of samples included 10 HC, 29 LC and 33 HCC cases. The HC liver samples were obtained from biopsy tissues, from which other common liver pathological changes were excluded. The LC sample was also obtained from biopsy tissue, and a pathologist confirmed the HCC sample via histological verification. The third set of samples included 37 HC, 36 CHB, 40 LC and 47 HCC cases. All patients were HBV-positive with the exception of the NSCLC patient. No significant differences were found between the study groups in terms of age or gender. The LC patients who were recruited for this study were excluded if they also suffered from autoimmune diseases and/or alcoholic cirrhosis. The blood samples were stored at 4°C before plasma separation, and the tissue samples were stored at -80°C until DNA extraction. More detailed clinical information is provided in Table 3.

**Table 3:** **Clinicopathological features of patient and healthy controls in the present study**

	Plasm methylome						Tissue qMSP			Plasm BSP		
	HC1	CHB2	LC3	HCC4	NSCLC5	HC1	LC3	HCC4	HC1	CHB2	LC3	HCC4
**Cases**	31	30	27	29	26	10	29	33	37	36	40	47
**Gender**												
**Male**	28 (90.3%)	27 (90.0%)	24 (88.9%)	26 (89.7%)	23 (88.5%)	6 (60%)	25 (86.2%)	27 (84.9%)	30 (81.1%)	28 (77.6%)	33 (78.8%)	39 (83.0%)
**Female**	3 (9.7%)	3 (10.0%)	3 (11.1%)	3 (10.3%)	3 (11.5%)	4 (40%)	4 (13.8%)	6 (15.1%)	7 (18.9%)	8 (22.2%)	7 (21.2%)	8 (17.0%)
**Age**	50.3 ± 8.6	50.7 ± 7.6	50.8 ± 9.6	55.8 ± 8.5	52.4 ± 9.6	42.2 ± 6.53	46.9 ± 9.2	50.3 ± 9.1	53.32 ± 5.3	52.53 ± 4.7	48.89 ± 10.7	54.29 ± 8.3
**HBV infection**												
**Yes**	0 (0%)	30 (100%)	27 (100%)	29 (100%)	0 (0%)	0 (0%)	30 (100%)	33 (100%)	0 (0%)	36 (100%)	40 (100%)	47 (100%)
**No**	31(100%)	0 (0%)	0 (0%)	0 (0%)	29 (100%)	10(100%)	0 (0%)	0 (0%)	37 (100%)	0 (0%)	0 (0%)	0 (0%)
**Differentiation**												
**Poor**		/	/	11 (37.9%)	12 (46.2%)	/	/	15 (45.5%)	/	/	/	22 (46.8%)
**Moderate**		/	/	10 (34.5%)	8 (30.7%)	/	/	13 (39.4)	/	/	/	15 (31.9%)
**High**		/	/	8(27.6%)	6 (23.1%)	/	/	5 (15.1%)	/	/	/	10 (21.3%)
**Tumor stage**												
**I/II**		/	/	14 (48.3%)	10 (38.5%)	/	/	13 (39.4%)	/	/	/	12 (25.5%)
**III/IV**		/	/	15 (51.7)	16 (61.5%)	/	/	20 (60.6%)	/	/	/	35 (74.5%)
**Tumor size**												
**≤3cm**		/	/	16 (55.2%)	/	/	/	11 (33.3%)	/	/	/	16 (34.1%)
**>3cm**		/	/	13 (44.8%)	/	/	/	22 (66.7%)	/	/	/	31 (65.9%)

In this study, group of patients were recruited by following criteria:

CHB (chronic HBV-related hepatitis) patient recruitment: more than 6 month history of diagnosed or suspected acute hepatitis; laboratory test evidence of HBV infection; mild to moderate increase of alanine aminotransferase (ALT) over years; no serious manifestations outside of hepatic system.

HC (healthy control) recruitment: individuals who attended routine healthy examinations; no histories of hepatitis; negative hepatitis B-related tests; no abnormal findings for hepatic biochemical tests.

HCC (HBV-related hepatocellular carcinoma) patient recruitment: pathological or clinical evidence indicating HCC diagnosis; chronic HBV related hepatitis or long-term HBV infection; normal or mildly abnormal liver biochemical examinations; no serious dysfunction in kidney, heart, lung, brain and other organs.

LC (HBV-related liver cirrhosis) patient recruitment: pathology or imaging diagnoses of cirrhosis patients; over a year of expected survival (liver function at level A or B by Child, MELD score ≤10); histories of chronic HBV infection or long-term hepatitis B infection; normal or mildly abnormal liver biochemical examinations; normal or slightly elevated serum AFP levels (≤20μg/L).

NSCLC (non-small cell lung cancer) patient recruitment: pathological or clinical diagnoses of lung cancer; no histories of hepatitis; no positive findings of HBV hepatitis infections by related blood tests; no abnormal findings in liver biochemical tests; no obvious dysfunctions in renal, heart, liver, brain or other organs.

Slash represents missing data.

### DNA and RNA extraction

A peripheral venous blood sample was drawn into an EDTA tube for each subject, and the plasma sample was separated within 2 hours. The plasma was isolated by centrifugation of the whole blood at 2,500 × g for 10 min. To eliminate potential cellular debris, an additional centrifugation step was performed at 15,000 × g for 10 min [34]. The cfDNA was extracted from 800-μl aliquots of plasma using the Qiagen Blood DNA Kit (QIAGEN, Hilden, Germany). The tissue DNA was isolated from 100 mg of corresponding tissues using a conventional proteinase K/organic extraction method as previously described [35]. The extraction of total RNA from liver tissue and reverse transcription were performed as described in reference [36].

### Processing of MethylCap-seq

To obtain adequate starting material for the MethylCap-seq library, 30 ng of plasma cfDNA from each participant were pooled for each group. Approximately 500 ng of DNA for each of the five libraries (HC, CHB, LC, HCC and NSCLC) was sonicated until all of the DNA fragments were within the desired size range (100 to 200 bp). The end-repair, adenosine base addition and adaptor ligation steps and methylated DNA enrichment were performed as previously described [37]. Finally, 1 μg of each product was placed in the Genome Analyzer II (Illumina, Inc., San Diego, CA) to generate 36 bp-long unpaired reads. The resulting data were processed to generate genomic methylation profiles and then uploaded to a public database (Gene Expression Omnibus: GSE54961 (2014)).

### Processing of MethylCap-seq data and GO analysis

We used the BWA alignment tool [38] with the default settings to map the 36-bp unpaired reads to the hg19 human genome reference assembly [39]. After removing the PCR duplicates with Picard (http://broadinstitute.github.io/picard/), SAMtools and Picard were used to convert, sort and index the aligned data. The DMRs in the four observed groups (CHB, LC, HCC and NSCLC) for comparison with the HC were identified using two methods, MACS [40] and BALM [41], to increase the power of detection for the MethylCap-seq analysis. Methylation peaks (hypermethylated regions) were identified using MACS for the four observed groups and HC, respectively, as previously described [37]. For BALM, a dual-threshold strategy was applied to decrease the false-positive detection of the DMRs. A high-confidence threshold of 0.975 for the four observed hypermethylated regions and a low-confidence threshold of 0.95 for the HC hypermethylated regions were set. Then, the specific methylation peaks of the four observed groups were defined as the hypermethylated regions. Similarly, HC-specific methylation peaks were defined as hypomethylated regions using the reverse setting. Whole-genome methylation (methylation of each CpG) was inferred with BALM, which was processed to perform Pearson correlation analyses among all of the samples using the R software. The refSeq genes (UCSC genes) and corresponding CpG islands (CGIs) were downloaded from the table browser of the UCSC database [39]. The BED files were manipulated with Bedtools [42] in addition to assorted Perl scripts.

### Screening with methylation-specific PCR (MSP) and validation with qMSP

The tissue DNA and cfDNA were converted with sodium bisulfite using an EpiTect Kit (QIAGEN, Hilden, Germany). All of the DNA (converted or unconverted) samples were immediately stored at -80°C for further analyses. Methylation-specific PCR (MSP) primer pairs were designed using online software (http://www.urogene.org/cgi-bin/methprimer/methprimer.cgi; Additional file 21: Table S1). In total, 40 MSPs were performed, and the results were visualized on gels. The MSP products were cloned by T-Vectors and verified by sequencing. The *in vitro* methylated DNA from the HepG2 cells was obtained using the CpG (M. SssI) methyltransferase (NEB, MA,USA) treatment and used as a positive control. Water was used as a non-template control. Up to 33 gene targets were measured using methylation-specific real-time PCR (qMSP) as previously described [43]. In brief, the PCR products of the methylated and unmethylated sequences of each gene target were cloned into pGEM-T Easy Vectors (Tiangen, Beijing, China). The plasmids were diluted, and a standard curve was constructed to measure both the methylated and unmethylated targets. Real-time PCR was conducted with the Rotor-Gene Q (QIAGEN, Valencia, CA). Target gene methylation was calculated using the following formula: 100 × methylated reaction/(unmethylated reaction + methylated reaction).

### Multiplex-BSP-seq

The BSP regions were situated within the regions that were assessed by the qMSP analysis. BSP amplicons that were 80 to 120 bp in size were obtained because of the fragmentation of the cfDNA template. The BSP primer pairs were designed using online software (http://www.urogene.org/cgi-bin/methprimer/methprimer.cgi; Additional file 19: Table S2). Degenerate primers were utilized in the promoter regions in case there where inevitably contained CpG sites. To overcome the difficulties that are associated with the scarcity of cfDNA, Multiplex-BSP-seq was performed. In total, 35 BSP primers were divided into three panels according to their annealing temperatures. Three independent multiplex-PCR amplifications were conducted for each cfDNA sample, and the three PCR products were combined in equal amounts as determined by gel electrophoresis and Qubit for ligation with a barcode adaptor (oligonucleotide sequences © 2007-2012 Illumina, Inc. All rights reserved). A final pooling, which contained equal quantities of each participant, was performed with all of the ligation products, and 12 cycles of PCR were performed using library amplification primers to produce the templates for deep sequencing. Illumina HiSeq 2500 was used to generate 100-bp paired-end reads (Additional file 22: Figure S1). The mapping of the raw reads was performed by Bismark (version 0.7.7) and Bowtie2 (version 2.0.5), and the resulting SAM data were sorted by SAMtools. The R methylKit package was utilized to assess the methylation levels at each CpG locus with default settings unless otherwise specified. The methylation level (%) that was measured at each individual CpG site was expressed as the percentage of methylated CpG versus unmethylated CpG.

### Real-time reverse transcription PCR

The expression levels of selected genes were measured by real-time quantitative RT-PCR analysis. Three or more reactions were performed for each sample using a RealMasterMix (SYBR Green) kit (TianGen, Beijing, China) with gene-specific primers (Additional file 23: Table S3) on a Rotor-Gene Q (QIAGEN, Valencia, CA). The RNA quantity was normalized to the ACTB content, and gene expression was quantified according to the 2^-ΔCt^ method.

### Statistical analyses

To evaluate the assay’s potential for discriminating between different disease stages, the data that were obtained for the HC, CHB, LC and HCC samples were used to calculated the optimal cut-off value using the most significant area under the receiver operating characteristic (ROC) curve. Univariate models were used to examine the association between the gene CpG methylation and HCC stage. Multivariate models were then developed that adjusted for the most important covariates, including age and gender. The measurement data were analyzed using a one-way ANOVA. The sample means were compared with an unpaired *t*-test, assuming unequal variances, and all of the tests were two-tailed. *P* values are shown for statistically significant findings. All of the statistical analyses were performed using the SPSS statistical package (version 13.0, Chicago, IL, USA).

## Electronic supplementary material

Additional file 1: Table S4: Data mining of plasma cfDNA MethylCap-seq libraries (1). (XLSX 9 KB)

Additional file 2: Table S5: Pairwise overlap of bases of peak regions for each sample as indicated by BALM and MACS. (XLSX 9 KB)

Additional file 3: Table S6: Data mining of plasma cfDNA MethylCap-seq libraries (2). *peaks are considered to be shared when they have overlapping regions. (XLSX 9 KB)

Additional file 4: Table S7: Data mining of plasma cfDNA MethylCap-seq libraries (3). (XLSX 9 KB)

Additional file 5: Table S8: General characteristics of aberrant methylation patterns according to UCSC genomic context definitions. (XLSX 8 KB)

Additional file 6: Table S9: Plasma cfDNA DMGs belong to early stage of HCC carcinogenesis progression. ※logarithmic transformations of *P* values to show significance levels of differentially methylated regions (for DMR estimations) or methylation blocks (for MACS). Higher values indicate higher probabilities of DMR or methylation blocks. (XLSX 26 KB)

Additional file 7: Table S10: Plasma cfDNA DMGs belong to middle stage of HCC carcinogenesis progression. (XLSX 29 KB)

Additional file 8: Table S11: Plasma cfDNA DMGs belong to late stage of HCC carcinogenesis progression. (XLSX 29 KB)

Additional file 9: Table S12: Early HCC development-associated DMGs in cfDNA as determined by genomic CGI involvement. (XLSX 25 KB)

Additional file 10: Table S13: Middle HCC development-associated DMGs in cfDNA as determined by genomic CGI involvement. (XLSX 26 KB)

Additional file 11: Table S14: Late HCC development-associated DMGs in cfDNA as determined by genomic CGI involvement. (XLSX 27 KB)

Additional file 12: Table S15: Early HCC development-associated DMGs in cfDNA as determined by genomic CGI-shore involvement. (XLSX 109 KB)

Additional file 13: Table S16: Middle HCC development–associated DMGs in cfDNA as determined by genomic CGI-shore involvement. (XLSX 106 KB)

Additional file 14: Table S17: Late HCC development-associated DMGs in cfDNA as determined by genomic CGI-shore involvement. (XLSX 81 KB)

Additional file 15: Table S18: Plasma cfDNA DMGs shared between HCC and NSCLC. (XLSX 42 KB)

Additional file 16: Table S19: Plasma cfDNA DMGs in HCC after deletion of NSCLC DMGs. (XLSX 46 KB)

Additional file 17: Table S20: Plasma cfDNA DMGs in NSCLC after deletion of HCC DMGs. (XLSX 22 KB)

Additional file 18: Figure S2: Tissue DNA screening and validation of hyper-DMRs in HCC development. MSP screening of 40 hyper-DMR targets in 30 HCC and 10 HC and qMSP validation of 33 hyper-DMR targets in same set of HCC and HC plus 29 LC. Row, the gene target investigated; column, the sample DNA; MSP, positive or negative category result. qMSP, continuous measurement result. (PDF 292 KB)

Additional file 19: Table S2: Primer sets of Multiplex-BSP-seq analysis of candidate genes.*CpG dinucleotides flanked by BSP primers were counted . # for degenerate primer. Y = C/T and R = A/G. (XLSX 13 KB)

Additional file 20: Table S21: Comparison of hypermethylation DMGs in plasma cfDNA from this study with hypermethylated CpG probe sites in HCC tissues that were reported by Shen *et al.* [15]. *differentially hypermethylated regions in HCC relative to HC. ※*P* values associated with relationship between DMRs in HCC cfDNA and CpG identities in 450,000 sites as reported by Shen *et al.* [15]. (XLSX 83 KB)

Additional file 21: Table S1: Primer sets for MSP and qMSP analyses of candidate genes. (XLSX 19 KB)

Additional file 22: Figure S1: The process of Multiplex-BSP-seq. (PDF 414 KB)

Additional file 23: Table S3: Primer sets for RT-PCR. (XLSX 9 KB)

## Abbreviations

AUC: area under the curve
cfDNA: plasma cell-free DNA
DMG: differentially methylated genes
DMR: differentially methylated regions
DNAm: DNA methylation
LC: liver cirrhosis
MSP: methylation-specific PCR
NSCLC: non-small cell lung cancer
OR: odds ratio.

## References

[1] Bruix J, Boix L, Sala M, Llovet JM (2004). Focus on hepatocellular carcinoma. Cancer Cell.

[2] Dufour JF, Johnson P (2010). Liver cancer: from molecular pathogenesis to new therapies: summary of the EASL single topic conference. J Hepatol.

[3] Tischoff I, Tannapfel A (2008). DNA methylation in hepatocellular carcinoma. World J Gastroenterol.

[4] Parkin DM, Bray FI, Devesa SS (2001). Cancer burden in the year. The global picture. Eur J Cancer.

[5] Zucman-Rossi J, Laurent-Puig P (2007). Genetic diversity of hepatocellular carcinomas and its potential impact on targeted therapies. Pharmacogenomics.

[6] Gehring AJ, Ho ZZ, Tan AT, Aung MO, Lee KH, Tan KC, Lim SG, Bertoletti A (2009). Profile of tumor antigen–specific CD8 T cells in patients with hepatitis B virus–related hepatocellular carcinoma. Gastroenterology.

[7] Huang J, Wang Y, Guo Y, Sun S (2010). Down-regulated microRNA-152 induces aberrant DNA methylation in hepatitis B virus-related hepatocellular carcinoma by targeting DNA methyltransferase 1. Hepatology.

[8] Vivekanandan P, Daniel HD, Kannangai R, Martinez-Murillo F, Torbenson M (2010). Hepatitis B virus replication induces methylation of both host and viral DNA. J Virol.

[9] Wei X, Xiang T, Ren G, Tan C, Liu R, Xu X, Wu Z (2013). miR-101 is down-regulated by the hepatitis B virus x protein and induces aberrant DNA methylation by targeting DNA methyltransferase 3A. Cell Signal.

[10] Letelier P, Brebi P, Tapia O, Roa JC (2012). DNA promoter methylation as a diagnostic and therapeutic biomarker in gallbladder cancer. Clin Epigenetics.

[11] Gao W, Kondo Y, Shen L, Shimizu Y, Sano T, Yamao K, Natsume A, Goto Y, Ito M, Murakami H, Osada H, Zhang J, Issa JP, Sekido Y (2008). Variable DNA methylation patterns associated with progression of disease in hepatocellular carcinomas. Carcinogenesis.

[12] Hernandez-Vargas H, Lambert MP, Calvez-Kelm F, Gouysse G, McKay-Chopin S, Tavtigian SV, Scoazec JY, Herceg Z (2010). Hepatocellular carcinoma displays distinct DNA methylation signatures with potential as clinicalpredictors. PLoS One.

[13] Shin SH, Kim BH, Jang JJ, Suh KS, Kang GH (2010). Identification of novel methylation markers in hepatocellular carcinoma using a methylationarray. J Korean Med Sci.

[14] Ammerpohl O, Pratschke J, Schafmayer C, Haake A, Faber W, von Kampen O, Brosch M, Sipos B, von Schönfels W, Balschun K, Röcken C, Arlt A, Schniewind B, Grauholm J, Kalthoff H, Neuhaus P, Stickel F, Schreiber S, Becker T, Siebert R, Hampe J (2011). Distinct DNA methylation patterns in cirrhotic liverand hepatocellular carcinoma. Int J Cancer.

[15] Shen J, Wang S, Zhang YJ, Kappil M, Wu HC, Kibriya MG, Wang Q, Jasmine F, Ahsan H, Lee PH, Yu MW, Chen CJ, Santella RM (2012). Genome-wide DNA Methylation Profiles in Hepatocellular Carcinoma. Hepatology.

[16] Um TH, Kim H, Oh BK, Kim MS, Kim KS, Jung G, Park YN (2011). Aberrant CpG island hypermethylation in dysplastic nodules and early HCC of hepatitis B virus-related human multistep hepatocarcinogenesis. J Hepatol.

[17] Ren N, Qin LX, Tu H, Liu YK, Zhang BH, Tang ZY (2005). Quantitative analysis of circulating DNA level in plasma from patients with hepatocellular carcinoma and its potential clinical value. J Fudan Univ Med Sci.

[18] Zhai R, Zhao Y, Su L, Cassidy L, Liu G, Christini DC (2012). Genome-wide DNA methylation profiling of cell-free serum DNA in esophageal adenocarcinoma and barrett esophagus. Neoplasia.

[19] Han H, Cortez CC, Yang X, Nichols PW, Jones PA, Liang G (2011). DNA methylationdirectly silences genes with non-CpG island promoters and establishes a nucleosome occupied promoter. Hum Mol Genet.

[20] Irizarry RA, Ladd-Acosta C, Wen B, Wu Z, Montano C, Onyango P, Cui H, Gabo K, Rongione M, Webster M, Ji H, Potash JB, Sabunciyan S, Feinberg AP (2009). The human colon cancer methylome shows similar hypo- and hypermethylation at conserved tissue-specific CpG island shores. Nat Genet.

[21] Doi A, Park IH, Wen B, Murakami P, Aryee MJ, Irizarry R, Herb B, Ladd-Acosta C, Rho J, Loewer S, Miller J, Schlaeger T, Daley GQ, Feinberg AP (2009). Differential methylation of tissue- and cancer-specific CpG island shores distinguishes humaninduced pluripotent stem cells, embryonic stem cells and fibroblasts. Nat Genet.

[22] Jemal A, Bray F, Center MM, Ferlay J, Ward E, Forman D (2011). Global cancer statistics. CA Cancer J Clin.

[23] Marcellin P, Gane E, Buti M, Afdhal N, Sievert W, Jacobson IM, Washington MK, Germanidis G, Flaherty JF, Schall RA, Bornstein JD, Kitrinos KM, Subramanian GM, McHutchison JG, Heathcote EJ (2012). Regression of cirrhosis during treatment with tenofovirdisoproxilfumarate for chronic hepatitis B: a 5-year open-label follow-up study. Lancet.

[24] Gaudet F, Hodgson JG, Eden A, Jackson-Grusby L, Dausman J, Gray JW, Leonhardt H, Jaenisch R (2003). Induction of tumors in mice by genomic hypomethylation. Science.

[25] Rakyan VK, Hildmann T, Novik KL, Lewin J, Tost J, Cox AV, Andrews TD, Howe KL, Otto T, Olek A, Fischer J, Gut IG, Berlin K, Beck S (2004). DNA methylation profiling of the human major histocompatibility complex: a pilot study for the human epigenome project. PLoS Biol.

[26] Sung WK, Lu Y, Lee CWH, Zhang D, Ronaghi M, Lee CG (2009). Deregulated Direct Targets of the Hepatitis B Virus (HBV)Protein, HBx, Identified through Chromatin Immunoprecipitation and Expression Microarray Profiling. J Biol Chem.

[27] Tao R, Li J, Xin J, Wu J, Guo J, Zhang L, Jiang L, Zhang W, Yang Z, Li L (2011). Methylation profile of single hepatocytes derived from hepatitis B virus-related hepatocellular carcinoma. PLoS One.

[28] Maria ND, Manno M, Villa E (2002). Sex hormones and liver cancer. Mol Cell Endocrinol.

[29] Fisel P, Kruck S, Winter S, Bedke J, Hennenlotter J, Nies AT, Scharpf M, Fend F, Stenzl A, Schwab M, Schaeffeler E (2013). DNA methylation of the SLC16A3 promoter regulates expression of the human lactate transporter MCT4 in renal cancer with consequences for clinical outcome. Clin Cancer Res.

[30] Nigam SK, Bush KT, Bhatnagar V (2007). Drug and toxicant handling by the OAT organic anion transporters in the kidney and other tissues. Nat Clin Pract Nephrol.

[31] Burckhardt G, Burckhardt BC (2011). In vitro and in vivo evidence of the importance of organic anion transporters (OATs) in drug therapy. Drug Transporters.

[32] Gou D, Wang J, Gao L, Sun Y, Peng X, Huang J, Li W (2004). Identification and functional analysis of a novel human KRAB/C2H2 zinc finger gene ZNF300. Biochim Biophys Acta.

[33] Wang T, Wang XG, Xu JH, Wu XP, Qiu HL, Yi H, Li WX (2012). Overexpression of the human ZNF300 gene enhances growth and metastasis of cancer cells through activating NF-kB pathway. J Cell Mol Med.

[34] Nygren AO, Dean J, Jensen TJ, Kruse S, Kwong W, Boom D, Ehrich M (2010). Quantification of Fetal DNA by Use of Methylation-Based DNA Discrimination. Clin Chem.

[35] Yu J, Zhang H, Ma Z, Lu W, Wang Y, Zhu JD (2003). Methylation profiling of twenty four genes and the concordant methylation behaviours of nineteen genes that may contribute to hepatocellular carcinogenesis. Cell Res.

[36] Doleshal M, Magotra AA, Choudhury B, Cannon BD, Labourier E, Szafranska AE (2008). Evaluation and validation of total RNA extraction methods for MicroRNA expression analyses in formalin-fixed. Paraffin-embedded tissue J Mol Diagn.

[37] Zhao Y, Guo S, Sun J, Huang Z, Zhu T, Zhang H, Gu J, He Y, Wang W, Ma K, Wang J, Yu J (2012). Methylcap-Seq reveals novel DNA methylation markers for the diagnosis and recurrence prediction of bladder cancer in a Chinese population. PLoS One.

[38] Li H, Durbin R (2009). Fast and accurate short read alignment with Burrows-Wheeler transform. Bioinformatics.

[39] Kuhn RM, Haussler D, Kent WJ (2013). The UCSC genome browser and associated tools. Brief Bioinform.

[40] Zhang Y, Liu T, Meyer CA, Eeckhoute J, Johnson DS, Bernstein BE, Nusbaum C, Myers RM, Brown M, Li W, Liu XS (2008). Model-based analysis of ChIP-Seq (MACS). Genome Biol.

[41] Lan X, Adams C, Landers M, Dudas M, Krissinger D, Marnellos G, Bonneville R, Xu M, Wang J, Huang TH, Meredith G, Jin VX (2011). High resolution detection and analysis of CpG inucleotides methylation using MBD-Seq technology. PLoS One.

[42] Quinlan AR, Hall IM (2010). BEDTools: a flexible suite of utilities for comparing genomic features. Bioinformatics.

[43] Lacopetta B, Grieu F, Philips M, Ruszkiewicz A, Moore J, Minamoto T, Kawakami K (2007). Methylation levels of LINE-1 repeats and CpG island loci are inversely related in normal colonic mucosa. Cancer Sci.

