# Supplementary figures and images for: Genome-wide methylation profiling of the different stages of hepatitis B virus-related hepatocellular carcinoma development in plasma cell-free DNA reveals potential biomarkers for early detection and high-risk monitoring of hepatocellular carcinoma
Source: Clin Epigenetics. 2014 Dec 2;6(1):30. doi: 10.1186/1868-7083-6-30 (PMC4391300; doi:10.1186/1868-7083-6-30)

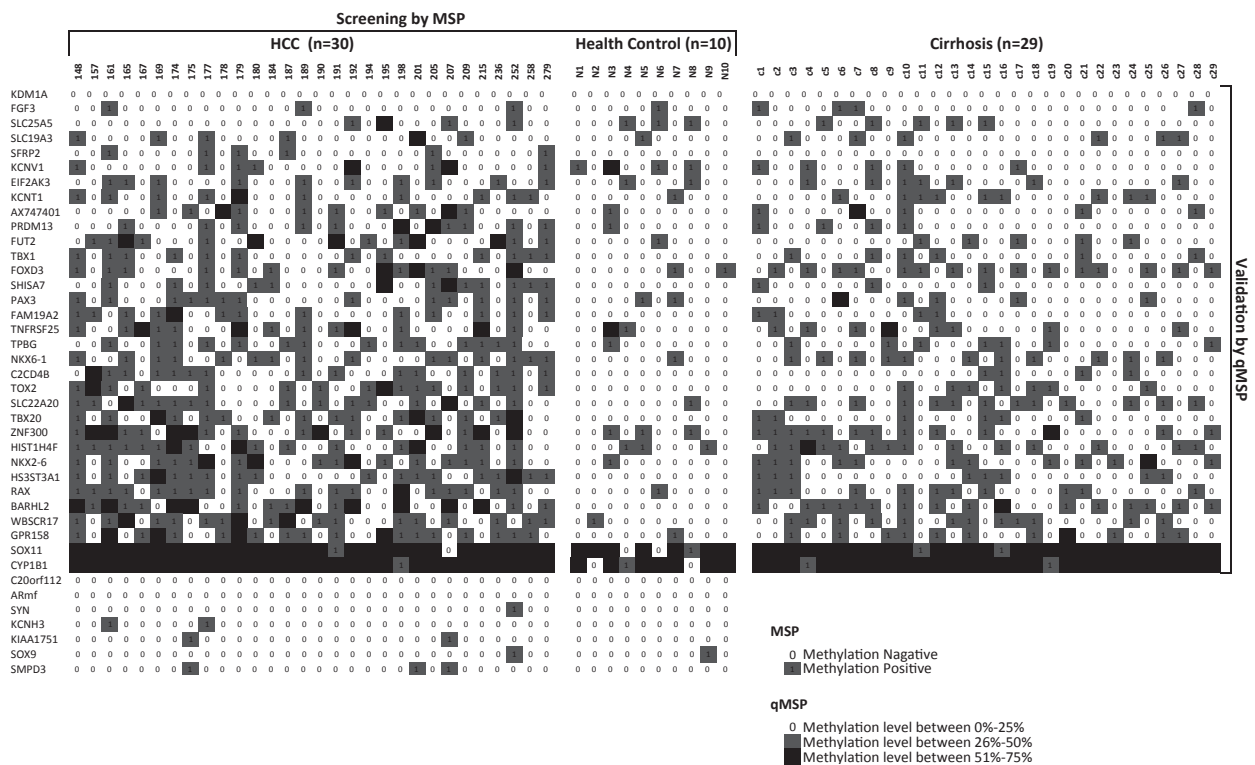

Supplement: Supplementary file 18 — Additional file 18: Figure S2: Tissue DNA screening and validation of hyper-DMRs in HCC development. MSP screening of 40 hyper-DMR targets in 30 HCC and 10 HC and qMSP validation of 33 hyper-DMR targets in same set of HCC and HC plus 29 LC. Row, the gene target investigated; column, the sample DNA; MSP, positive or negative category result. qMSP, continuous measurement result. (PDF 292 KB) [file 13148_2014_94_MOESM18_ESM.pdf]

Supplementary Figure 2

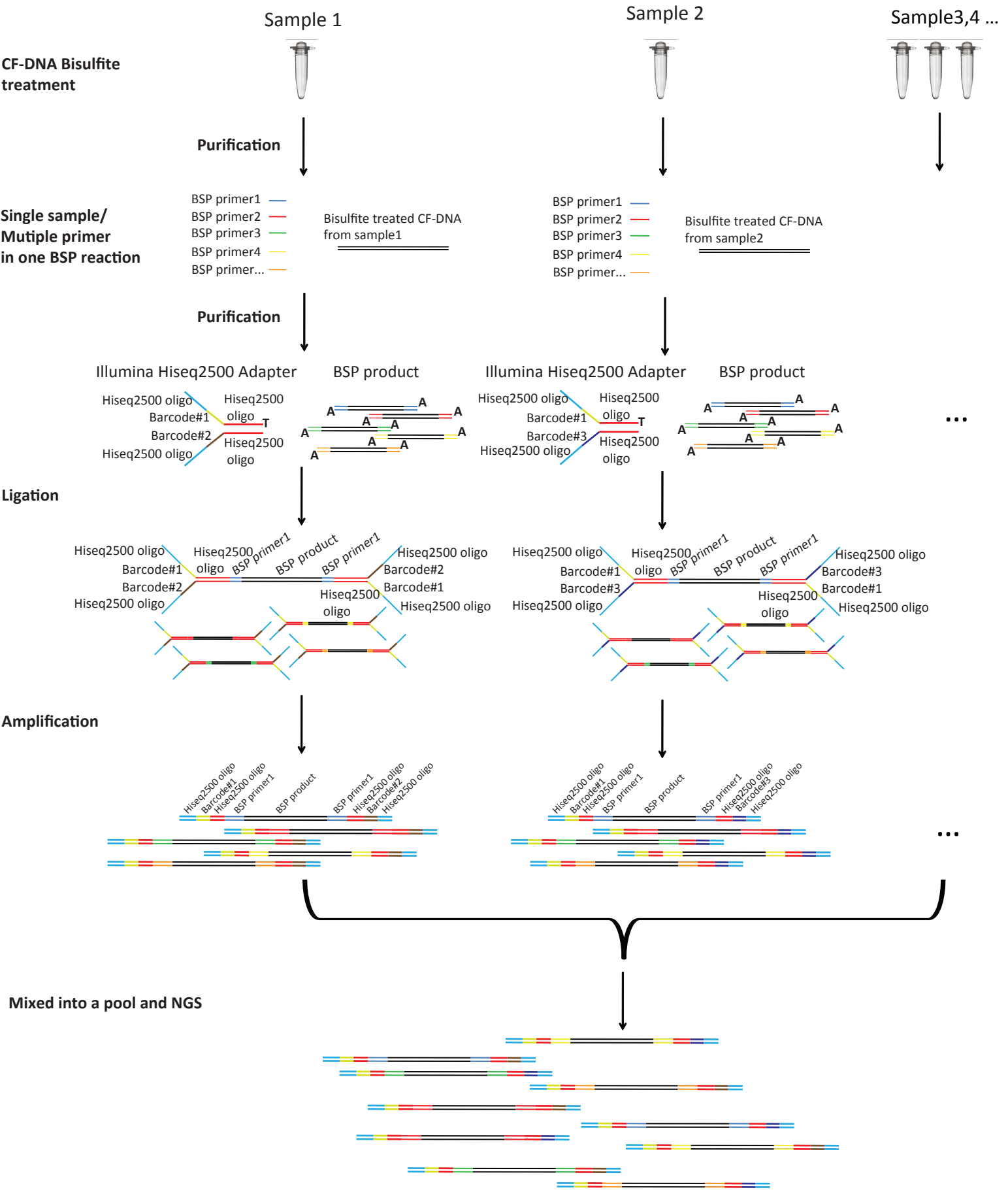

Supplement: Supplementary file 22 — Additional file 22: Figure S1: The process of Multiplex-BSP-seq. (PDF 414 KB) [file 13148_2014_94_MOESM22_ESM.pdf]
